# An enhanced genetic-based multi-objective mathematical model for industrial supply chain network

**DOI:** 10.1371/journal.pone.0315545

**Published:** 2025-03-04

**Authors:** Yanchun Li

**Affiliations:** Department of Basic Sciences, Jilin University of Architecture and Technology, Changchun, Jilin, China; Chang Gung University, TAIWAN

## Abstract

The multi-objective supply chain needs a full look at enterprise costs, coordinated delivery of different products, and more fluidity and efficiency within the network of the supply chain. However, existing methodologies rarely delve into the intricacies of the industrial supply chain. Therefore, in the emerging industrial supply chain network, a model for the multi-objective problem was made using a meta-heuristic approach, specifically the improved genetic algorithm, which is a type of soft computing. To create the initial population, a hybrid approach that combines topology theory and the random search method was adopted, which resulted in a modification of the conventional single roulette wheel selection procedure. Additionally, the crossover and mutation operations were enhanced, with determining their respective probabilities determined through a fusion of the elite selection approach and the roulette method. The simulation results indicate that the improved genetic algorithm reduced the supply load from 0.678 to 0.535, labor costs from 1832 yuan to 1790 yuan, and operational time by approximately 39.5%, from 48 seconds to 29.5 seconds. Additionally, the variation in node utilization rates significantly decreased from 30.1% to 12.25%, markedly enhancing resource scheduling efficiency and overall balance within the supply chain.

## 1 Introduction

The development of modern supply chains has become a national strategic priority in China, aiming to accelerate the country’s economic growth through robust supply-side structural reforms [1]. The optimization of supply chain operations is vital for minimizing costs and maximizing social benefits. However, the growing complexity of the global economic landscape presents significant challenges to maintaining the smooth operation, security, stability, and efficiency of industrial supply chains. In response to these challenges, multi-objective optimization has emerged as a crucial approach for modeling and optimizing various parameters simultaneously to achieve a balanced trade-off between conflicting objectives. Despite the increasing application of multi-objective optimization techniques in supply chain management, there remains a limited focus on their integration with industrial supply chain networks, particularly in utilizing advanced multi-objective mathematical modeling and meta-heuristic algorithms.

While existing studies have addressed multi-objective supply chain optimization using time-based relationships to enhance economic efficiency [2], the methodologies employed often lack the necessary specificity to address the unique demands of industrial supply networks. For instance, traditional approaches may not fully capture the dynamic interdependencies within the supply chain, leading to suboptimal solutions. Mathematical modeling offers a powerful tool for solving practical supply chain problems by formulating mathematical representations of complex processes [3]. Nonetheless, there is a noticeable gap in the application of multi-objective mathematical modeling techniques combined with meta-heuristic algorithms tailored for the industrial supply domain. Specifically, limited research has explored the use of genetic algorithms (GAs) for optimizing multi-objective industrial supply chain problems, leaving room for further innovation and refinement in this area.

Multi-objective supply chain problems typically involve optimizing several conflicting objectives simultaneously [4–6]. Key challenges include cost optimization, which entails minimizing production, transportation, and inventory costs while meeting demand and maintaining service quality. Coordinated delivery is another critical issue, requiring the optimization of logistics and distribution processes to ensure timely and efficient delivery of various products. Enhancing supply chain efficiency is also paramount, focusing on improving resource allocation, reducing idle times, and maintaining smooth operational performance. Additionally, balancing multiple objectives such as cost reduction versus service improvement, or efficiency versus flexibility, poses a significant challenge in multi-objective supply chain management. Despite the limited research on genetic algorithms in multi-objective optimization for supply chains, the adaptability of GA’s problem-solving methods to this domain is noteworthy.

Meta-heuristic algorithms, which include methods such as tabu search [7], simulated annealing [8], and GAs [9–11], are well-suited for exploring optimal solutions in complex multi-objective problems. GAs, in particular, stand out due to their adaptive search capabilities and their ability to simulate the process of natural evolution to identify optimal solutions across diverse fields such as operations management [12], financial analysis [13], and medicine [14]. Nevertheless, their application in multi-objective supply chain optimization remains underexplored. Existing studies tend to apply GAs to broader optimization contexts, often without specific modifications to cater to the particularities of industrial supply chains, which are characterized by diverse product requirements, fluctuating demand, and intricate logistical networks. This gap in tailored algorithmic approaches highlights the need for an improved GA that can more effectively address these distinct challenges.

The current research aims to bridge this gap by developing an advanced meta-heuristic model for multi-objective optimization in industrial supply chains. The primary goals are to tackle key issues such as cost minimization, coordinated delivery, and overall supply chain fluidity. The novelty of this research lies in the introduction of a hybrid initialization approach for the genetic algorithm, combining topology theory with random search methods to enhance the diversity of the initial population and improve optimization outcomes. Additionally, the study focuses on resolving the multi-objective balance optimization problem through a refined genetic algorithm, offering a practical and robust solution that better aligns with the operational demands of industrial supply chains.

Main contributions are as follows:

(1) Development of an Advanced Meta-Heuristic Model: This research presents a novel model for multi-objective optimization in industrial supply chains, utilizing an improved genetic algorithm specifically tailored for addressing the complexities of this domain. The model represents a significant advancement in soft computing approaches and enhances existing optimization methodologies.
(2) Hybrid Initialization Approach: A new method that integrates topology theory with random search techniques is employed to generate a diverse and effective initial population for the genetic algorithm. This approach modifies traditional roulette wheel selection, leading to better optimization performance.
(3) Resolution of Multi-Objective Balance Optimization: By effectively resolving the multi-objective balance optimization problem, the research provides a practical and robust solution that enhances the efficiency and effectiveness of industrial supply.

Section 2 elaborates on the multi-objective balance optimization problem and the current state of research on genetic algorithms. Section 3 presents multi-objective supply chain modeling based on the improved genetic algorithm. Section 4 encompasses experiments and analysis, while Section 5 offers a summary and discusses potential limitations.

## 2 Related work

In the formulation of emerging industrial supply chains, our research primarily revolves around the multi-objective equilibrium optimization problem. Within the context of supply chains, this optimization problem pertains to the efficient allocation of resources such as nodes, manpower, and supply time to ensure optimal levels of various product deliveries. By achieving this balance, we aim to minimize resource consumption while enhancing the overall efficiency and fluidity of the supply chain.

**Fig 1:**
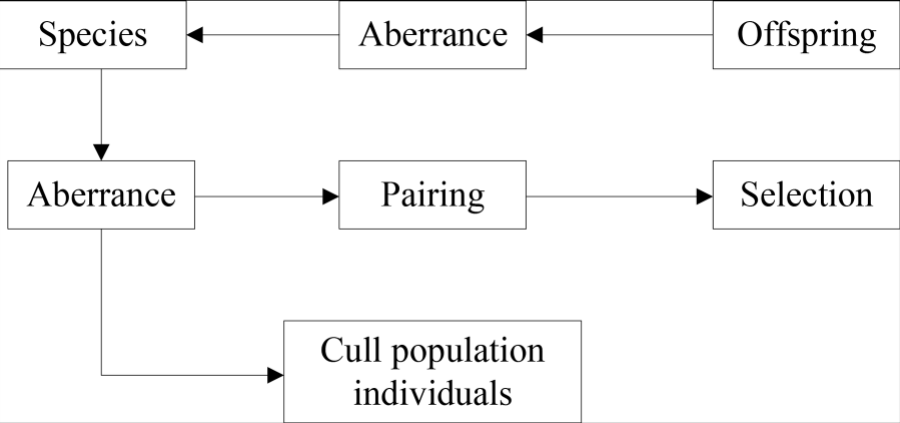
Natural selection diagram.

GA employ principles of natural selection and genetic theory to solve problems [15–17]. Fig 1 illustrates the flow chart depicting the process of natural selection. In GA, the chromosome corresponds to the encoding of the solution in problem-solving. Each component of the gene represents specific characteristics of the solution, and the characteristic values of individuals are interconnected. Crossing refers to the process of individuals mating, thereby generating another set of solutions.

In the realm of multi-objective optimization, the Non-dominated Sorting Genetic Algorithm II (NSGA-II) has emerged as a cornerstone due to its efficient and elitist approach. Introduced by Deb et al., NSGA-II leverages non-dominated sorting and crowding distance mechanisms to generate a diverse and high-quality set of solutions. This algorithm is renowned for its ability to manage conflicting objectives and deliver a well-distributed Pareto front, significantly enhancing solution accuracy and convergence [18]. Despite its advantages, NSGA-II’s computational complexity can be a limitation in large-scale problems, where its efficiency may decrease due to the extensive sorting and crowding distance calculations. Particle Swarm Optimization (PSO) is another meta-heuristic widely used for multi-objective optimization. The bi-objective web service composition algorithm designed for multi-cloud environments exemplifies PSO’s application in minimizing costs and enhancing security [19]. PSO’s adaptability and ease of implementation make it a popular choice; however, its performance heavily depends on parameter tuning and the diversity of the swarm, which can affect the convergence speed and solution quality in complex scenarios. This highlights a trade-off between algorithm simplicity and solution precision. The reliance on parameter settings often leads to a trade-off between algorithm simplicity and solution precision, necessitating the development of adaptive parameter adjustment techniques to improve PSO’s robustness.

Grey Wolf Optimization (GWO) has been successfully applied to multi-objective problems, offering robust solutions in various domains. For instance, Salimian et al. [20] presented a GWO-based model addressing cost and scheduling issues in IoT environments, while Guerrero [21]explored a genetic-based optimization approach for Quality of Service improvement. Although GWO exhibits good convergence properties, it struggles to maintain diversity in the Pareto front, particularly in high-dimensional solution spaces. Addressing this issue requires integrating diversity-preserving strategies to ensure comprehensive exploration and exploitation of the search space in high-dimensional problems. GA have also been instrumental in addressing multi-objective optimization challenges. For example, Behera et al., investigated a GA-based approach for optimizing task scheduling [22]. Another significant contribution from Saxena et al., which utilizes a GA-based method to balance communication costs and resource allocation [23]. While GA offers flexibility and adaptability to various problem structures, its performance can be inconsistent depending on the genetic operators used and the problem’s complexity, potentially affecting convergence and solution diversity.

In analyzing these meta-heuristic approaches, it is clear that each has its strengths and limitations. NSGA-II excels in generating diverse solutions but may face computational challenges with large problem sizes. PSO is valued for its simplicity and adaptability but requires careful parameter tuning. GWO provides robust solutions but can struggle with diversity maintenance in complex problems. GA offers flexibility but may show inconsistent performance depending on genetic operators and problem complexity. Integrating these approaches or developing hybrid methods could address some of these limitations, enhancing solution quality and algorithm efficiency in multi-objective optimization tasks. This comparative analysis underscores the importance of choosing the appropriate algorithm based on the specific requirements and constraints of the problem at hand.

Similarly, Satrio et al. [24] selected parameters such as thermostat settings, passive solar design, and cooler control as decision factors, using an artificial neural network in conjunction with a multi-objective GA to reduce the energy consumption of air conditioning systems in buildings.

Owais et al. [25] applied a multi-objective GA to solve the traffic network design problem. They utilized the GA as a comprehensive multi-objective algorithm that generates routes from scratch, ultimately yielding an efficient public transportation network.

In the context of multi-objective optimization in supply chain management, the types of parameters differ slightly from those in other fields. It is necessary to consider factors such as time cost, labor cost, and financial cost. Consequently, it becomes essential to develop a proprietary algorithm and implement appropriate feature construction to effectively address these requirements. The reviewed papers reveal a clear research gap in the application of GA to multi-objective optimization problems specific to supply chain management. While studies such as those by May et al. [26] and Li et al. [27] demonstrate the effectiveness of multi-objective GAs in fields like energy consumption and fuel cell optimization, they do not address the unique challenges posed by supply chain management. These challenges include the need to balance multiple, often conflicting objectives such as time, labor, and financial costs. The existing research, including work by Lamini et al. [28], and Rahmani et al. [29], focuses primarily on single-objective or generalized multi-objective problems but lacks a detailed exploration of how GAs can be specifically tailored to manage the diverse and interrelated parameters of supply chain optimization. Therefore, there is a notable gap in developing proprietary GA-based algorithms that address the complexities and specificities of supply chain management, suggesting a crucial area for further research.

Several issues arise from the limitations observed in existing algorithms. First, there is a need for more efficient diversity-preserving techniques that can maintain a well-distributed Pareto front, especially in high-dimensional and large-scale problems. Secondly, parameter tuning remains a critical challenge, particularly for algorithms like PSO, where performance is highly dependent on parameter settings. Thirdly, the integration of adaptive mechanisms in GAs to enhance the exploration and exploitation capabilities across various problem complexities warrants further investigation. Lastly, most algorithms lack scalability, which restricts their application to real-world problems involving large datasets and high computational demands. Addressing these issues through the development of hybrid models or algorithmic improvements is essential for advancing the field.

While existing research on multi-objective optimization has focused on generalized applications such as energy consumption, network design, and IoT, there is a notable lack of attention to the specific multi-objective challenges in supply chain management. Supply chain optimization presents unique difficulties, requiring the balancing of multiple, often conflicting objectives like cost minimization, coordinated delivery, and overall supply chain fluidity. These objectives are not independent; they are highly interdependent, meaning that improving one aspect (e.g., reducing costs) can have adverse effects on others (e.g., delivery speed or quality), making the optimization process more complex than in other domains. Current algorithms like NSGA-II, PSO, and GWO struggle to address the unique needs of supply chains due to the intricate interdependencies and the scale of real-world problems in this field. Most approaches are designed with a general-purpose mindset, lacking the tailored mechanisms required for handling the specific trade-offs and constraints found in industrial supply chains. Challenges such as scalability, diversity preservation in high-dimensional solutions, and effective parameter tuning further limit the applicability of existing methods in large, dynamic supply chain environments.

Therefore, there is a significant gap in developing algorithms that can account for the diverse and interconnected parameters characteristic of supply chain optimization. This gap includes the need for approaches that can manage the complexities of multi-objective trade-offs while providing robust, scalable solutions that align with industrial requirements. Addressing this gap requires algorithms that are not only efficient but also specifically designed to tackle the unique aspects of supply chain management.

## 3 Multi-objective supply chain model based on GA

**Fig 2:**
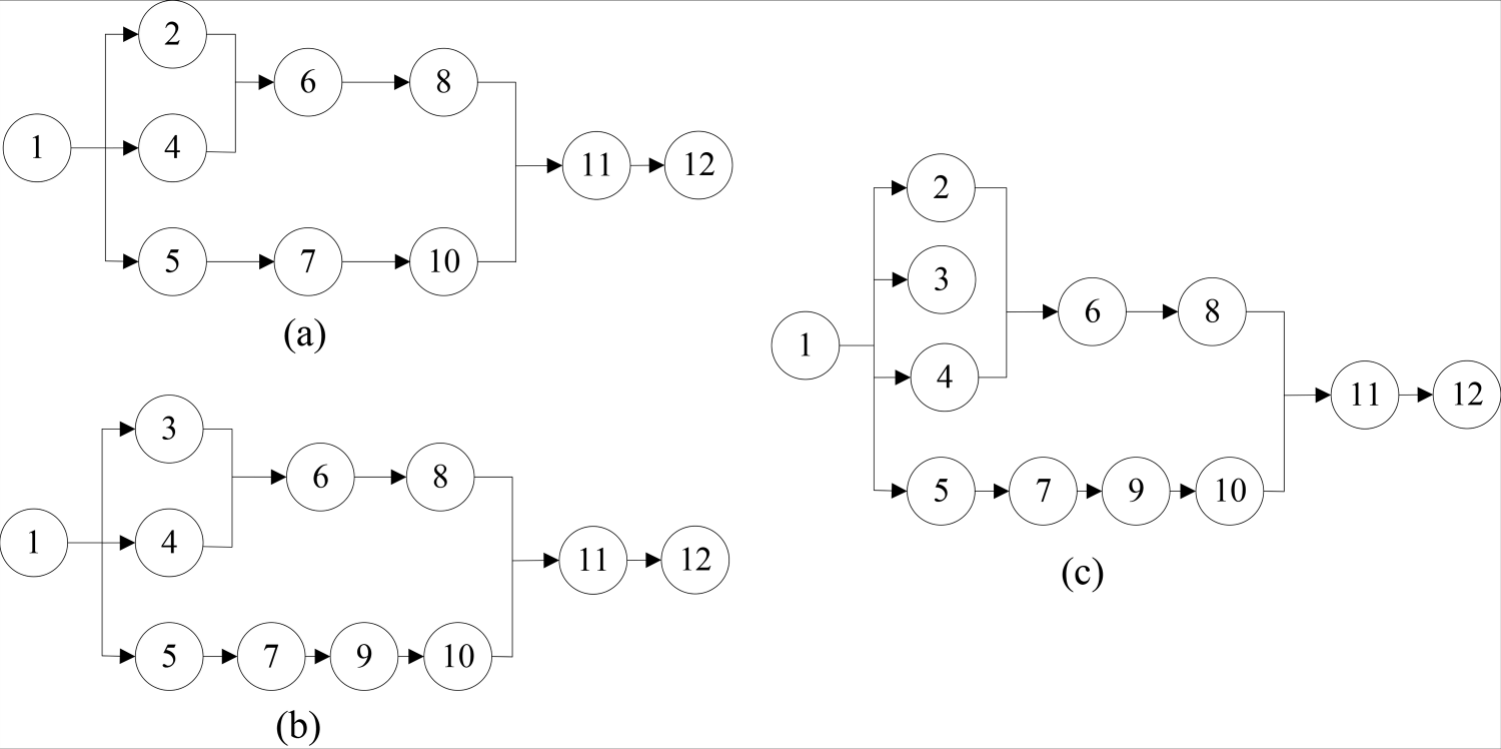
Product supply sequence diagram.

The multi-objective supply chain encompasses a diverse range of products within a single supply and demand network, wherein each supply node plays a crucial role in the transportation process. However, due to variations in the preparation processes associated with different product types, the issue of achieving a balanced multi-objective supply arises. The objective of balancing a multi-objective supply chain involves the rational allocation of all supply tasks among the supply nodes, while adhering to specific constraints. This allocation aims to ensure that the working hours of each parallel supply line are comparable, thereby maximizing the efficiency of the supply chain. To visually depict the balanced nature of a multi-objective supply chain, we have included a multi-objective supply sequence diagram in Fig 2.

The supply sequence requires that different types of products should be matched strictly according to the demand, combined through a series of operations, and finally a comprehensive multi-objective supply diagram is formed. In Fig 2, (a) and (b) respectively show the supply order of two products, and (c) shows the comprehensive product supply order formed by the combination of (a) and (b).

### 3.1 Problem description

In a multi-objective supply chain system, various products traverse through multiple supply nodes in a network. The goal is to balance the supply chain by managing different types of products, each with unique preparation processes. The balancing problem is to allocate all supply tasks across the supply nodes under certain constraints, ensuring that the work time on each parallel supply line is as similar as possible to maximize overall supply efficiency.

Objective 1 Minimize Total Supply Cost:


$$\text{min}\sum_{i=1}^{N}\sum_{j=1}^{M}C_{ij}\cdot x_{ij}$$

Where $C_{ij}$ is the cost of transporting product *i* from node *j*, and $x_{ij}$ is a binary variable indicating whether product *i* is assigned to node *j*.

Objective 2 Minimize Supply Line Imbalance with Penalty for Delay:


$$\text{min}\underset{j}{\text{max}}\left[\left(T_{j}-\text{mean}\left(T\right)\right)+\lambda \cdot {\left(T_{j}-D_{j}\right)}^{+}\right]$$

Where $T_{j}$ is the total processing time on supply line $j,\text{mean}\left(T\right)$ is the average processing time, $D_{j}$ is the desired processing time for line *j*, and *λ* is a penalty coefficient. The term ${\left(T_{j}-D_{j}\right)}^{+}$represents the positive deviation from the desired time.

Objective 3 Maximize Resource Utilization Efficiency:


$$\text{max}\left(\overset{M}{\underset{k=1}{\sum }}\frac{U_{k}}{R_{k}}\cdot \theta_{k}\right)$$

Where $U_{k}$ is the utilization rate of resource $k,R_{k}$ is the total capacity of resource *k*, and $\theta_{k}$ is a weight factor reflecting the importance of resource *k*.

### 3.2 GA coding and decoding

**Fig 3:**
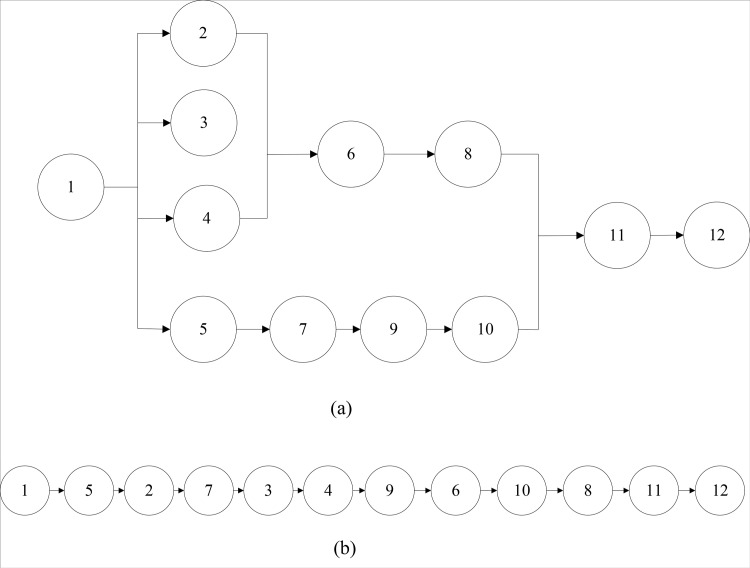
(a) Supply sequence chart; (b) The genetic code for the supply tasks.

Genetic algorithm is a meta-heuristic algorithm, which is one of the commonly used techniques in soft computing. As the starting point of GA, coding plays an irreplaceable role and is the key step to complete the whole design. The genetic code needs to be adapted to different tasks and different data requirements. Thus, the different coding methods have great influence on the later operation. Aiming at the multi-objective supply chain balance problem, according to the product supply sequence that has become a pattern, the products in the supply chain are coded by real number. As shown in Fig 3(a), according to the supply order priority relationship. Task N can be sequenced linearly, represented by 12 positions on the chromosome, as illustrated in Fig 3(b). Each job task corresponds to a gene’s position on the chromosome, and they are sequentially assigned to different supply nodes. Furthermore, it must be ensured that the cumulative production time within the supply chain consistently maintains a level below the supply cycle time.

After gene coding, the initial population needs to be caused. The initial population is a population set, which contains some preliminary solutions, so that the algorithm can operate on individuals in the population. When determining the population size, it should steer clear of selecting a size that is too small. Only by opting for a sufficiently large population size can we ensure an ample pool of methods to tackle the optimization problem and substantially increase the likelihood of finding the optimal solution. Indeed, a larger population size contributes to a more extensive exploration of possibilities, reducing the risk of getting trapped in local optima. In this study, when arranging genes on each chromosome within the population, strict adherence to the product supply order priority diagram is ensured to maintain the integrity and effectiveness of the optimization process.

Accordingly, this paper employs topological sorting theory and the random search algorithm to create the initial population. The operation steps are as follows:

Step 1: Assume that the product supply task set in the supply chain is $T_{1}=\{t_{1},t_{2},...,t_{i}\}$ , and the provisioning task can be expressed as i=1,2,3,...,n;
Step 2: According to the priority diagram of supply tasks, Select any task from set *T* that has no prior task or has been assigned prior task and put it into set $T_{1}$; At the same time, delete the supply order related to the selected supply task in the priority relation diagram;
Step 3: Find the corresponding gene position from any supply task in set $T_{1}$, and place it;
Step 4: Determine the set of supply tasks to be carried out. $T_{1}=\{t_{1},t_{2},...,t_{i}\}=\phi$, the product to be supplied has been assigned to the node, otherwise, the second step needs to be repeated.

To explain the whole process of population generatio, the job task relationship diagram in Fig 1 is taken as the research object, and its selected task set is $T_{1}=\{1,5,3,4,7,2,9,6,10,8,11,12\}$. The detailed process is shown in Table 1.

**Table 1:** The genetic allocation process of the initial population.

Gene location	Supplied task	$T_{1}$	selected task
1		1	1
2	1	2,3,4,5	5
3	1,5	2,3,4,7	3
4	1,5,3	2,4,7	4
5	1,5,3,4	2,7	7
6	1,5,3,4,7	2,9	2
7	1,5,3,4,7,2	6,9	9
8	1,5,3,4,7,2,9	6,10	6
9	1,5,3,4,7,2,9,6	8,10	10
10	1,5,3,4,7,2,9,6,10	8,11	8
11	1,5,3,4,7,2,9,6,10,8	11	11
12	1,5,3,4,7,2,9,6,10,8,11	12	12

The final chromosome coding sequence is {1,5,3,4,7,2,9,6,10,8,11,12}. Using topological ordering theory, genes can finally be arranged and positioned according to the actual supply order; To expand the search range of the algorithm, the random search method is adopted to expand the population number and improve the quality of the solution algorithm.

When calculating the fitness value of chromosomes, firstly, the encoded chromosomes are segmented according to the requirements of optimization objectives, and the supply tasks assigned by each node are obtained. The cycle time of supply in the first kind of supply chain balance problem CT is known, while the cycle time of supply in the second kind of supply chain balance problem $CT^{*}$ is unknown. Thus, one $CT^{*}$ can be assumed, and then calculated with a certain potential increment. The specific operation process is as follows.

Step 1: Set the initial supply cycle time, that is $CT_{theory}=t/n$;
Step 2: Take $CT^{*}$ as the cycle time, arrange the supply tasks reasonably in each node based on the work sequence priority diagram, if the time of each work place $t_{n}\leq CT^{*}$, then $CT^{*}$ is the minimum supply beat after gene sequencing, and the iterative process needs to be stopped, but when $t_{n}\geq CT^{*}$, it needs to continue the next Step;
Step 3: Complete the calculation of the potential increment of nodes in the actual supply chain $\Delta_{1},\Delta_{2},...,\Delta_{n}$;
Step 4: Let $CT=\max(t_{k})$, $CT^{*}=\min\{T_{k}+\Delta_{n}\}$, if $CT\leq CT^{*}$, then CT is the minimum cycle time in the supply chain, the search stops, otherwise, return to Step 2.

### 3.3 Fitness function

The fitness function is crucial for evaluating the quality of chromosomes within a population. It also serves as a tool to select the most promising individual as the approximate best candidate. During the selection process, individuals with high fitness values are retained and propagated to the succeeding generation, ensuring the continuation of advantageous traits and enhancing the overall performance of the population.

When designing the fitness function, especially in comparison to genetic coding, it is essential to consider the various relationships and data forms within the supply chain. Four objectives are typically considered simultaneously: the number of nodes, the actual supply cycle time, the processing cost of products, and the supply load balance of each node.

Due to the differing quantization scales and units of these objectives, numerical values can vary significantly. To address this issue, data normalization is required, transforming the data to lie within the interval of 0 to 1. Linear function normalization is employed to process all data points in the supply chain, ensuring a more standardized input for the optimization process.

The linear function normalization formula is shown in Formula (1):


$$X_{norm}=\frac{X-X_{\min}}{X_{\max}-X_{\min}}$$ (1)

All four objective functions are minimized, so the mathematical model of objective function is used as fitness function. The fitness function consists of four parts, as shown in Formula (2):


$$Fit=Fit_{1}+Fit_{2}+Fit_{3}+Fit_{4}$$ (2)

The number of nodes is optimized as shown in Formula (3):


$$Fit_{1}=Ceil\left(\frac{\sum_{k=1}^{K}m_{k}\sum_{i=1}^{1}t_{ki}}{M\times CT_{theory}}\right)$$ (3)

The optimization of the actual supply cycle time of the supply chain is shown in Formula (4):


$$Fit_{2}=\min\left(\sum_{i=1}^{I}\sum_{k=1}^{K}q_{k}x_{in}t_{ki})\right)$$ (4)

The optimization of the processing cost of products in the supply chain is shown in Formula (5):


$$Fit_{3}=\sum_{n=1}^{N}\sum_{d=1}^{D}\sum_{i=1}^{I}\left(C_{di}y_{dn}x_{in}\right)$$ (5)

The supply load optimization of each node in the supply chain is shown in Formula (6):


$$Fit_{4}=\sqrt{\frac{{\sum_{n=1}^{N}\left(\sum_{k=1}^{K}\sum_{i=1}^{I}q_{k}x_{in}t_{ki}-\sum_{n=1}^{N}\sum_{k=1}^{K}\sum_{i=1}^{I}q_{k}x_{in}t_{ki}/N\right)}^{2}}{N}}$$ (6)

### 3.4 Improved crossover mode

The crossing mode selected here is two-point crossing, and the operation steps are as follows.

**Fig 4:**
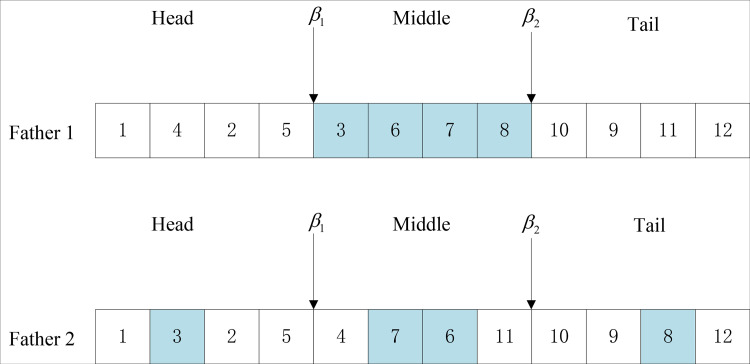
Selection of crossing point.

Step 1: Randomly select any two chromosomes (parent 1, parent 2) in the population as gene strings composed of N genes to cross:
Step 2: Randomly select two intersection points with parent 1 and parent 2 objects. $\beta_{1},\;\beta_{2}$, which meets $1<\beta_{1}<\beta_{2}<n$;
Step 3: Remove the middle part of the first generation, and arrange the genes according to the order of the second generation, and finally put the rearranged gold and silver back into the first generation; Likewise, remove the middle part of parent 2 and arrange the genes in the same order as those in Parent 1, and put the rearranged genes back into Parent 2 as well. The specific steps of crossing two points are shown in Fig 4.

**Fig 5:**
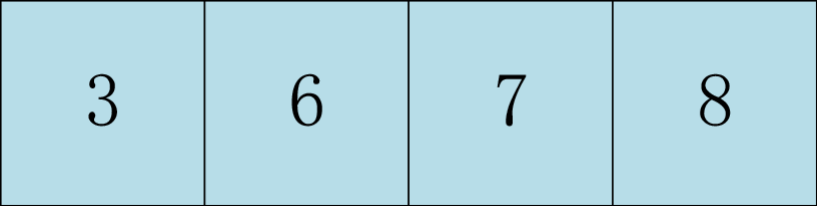
The sequence of genes in the middle part of father 1.

Two intersections were randomly selected in the parent 1, and the genes in the middle part were selected. The middle part of the parent 1 is shown in Fig 5.

**Fig 6:**
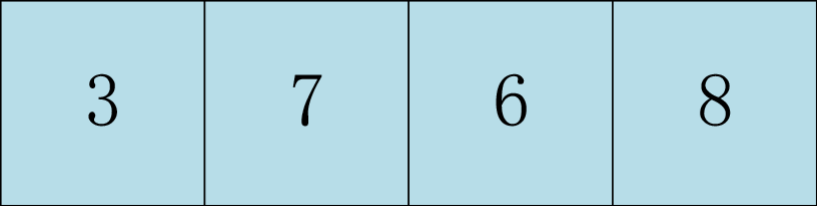
Alternative gene sequence in the middle part of father 1.

Find the arrangement order of genes 3, 6, 7, 8 from the parent 2, and take the arranged gene order as the middle part of the parent 1 as shown in Fig 6.

**Fig 7:**
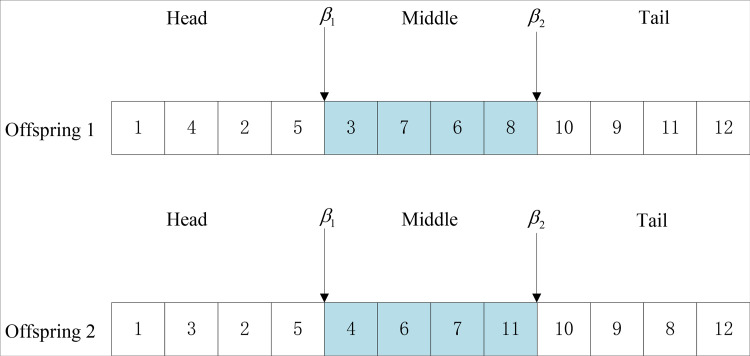
Crossed offspring chromosomes.

Chromosomes of progeny 1 and progeny 2 after two-point crossing are shown in Fig 7.

The use of roulette wheel selection for both crossover and mutation introduces a more nuanced approach to individual selection based on fitness levels, contrasting with the static probability approach of traditional GAs. In conventional GAs, crossover probability is typically set as a fixed value, often between 0.6 and 0.9, which may not yield the best results since the optimal probability can vary depending on the problem characteristics and the population state. In this study, the elite selection method ensures that the top-performing individuals, referred to as elite members, are retained in each generation. These elite individuals are given a higher probability of being selected for crossover based on their fitness values, thus increasing the likelihood of preserving superior traits in the offspring. The roulette wheel method is employed to select individuals for crossover by assigning selection probabilities proportional to their relative fitness within the population, thereby balancing exploration (genetic diversity) and exploitation (refinement of the best solutions).

### 3.5 Mutation

The mutation operation steps of enhanced GA algorithm are as follows.

Step 1: Select a chromosome as the parent chromosome randomly;
Step 2: Select a mutation point on the parent chromosome randomly;
Step 3: Remove the gene preceding the mutation point, regenerate a segment of genes, and ultimately obtain an entirely novel chromosome.

Select one parent 1 in Fig 3 for mutation operation, and follow the above mutation steps to explain.

**Fig 8:**
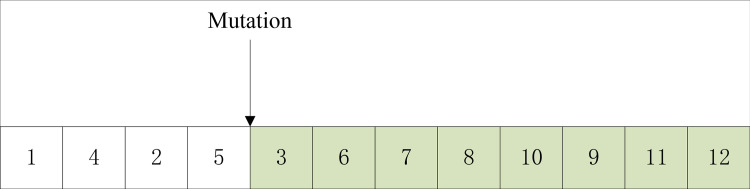
Recombinant gene map.

The four gene positions are reduced as a mutation point. The genes in the first four positions of the mutation point remain unchanged, and the eight genes after the mutation point are combined according to the actual operation sequence relationship to form an alternative chromosome, as shown in Fig 8.

An alternative chromosome was obtained by recombination, and its gene sequence was expressed as: 1-4-2-5-3-7-6-9-8-10-11-12.

**Fig 9:**
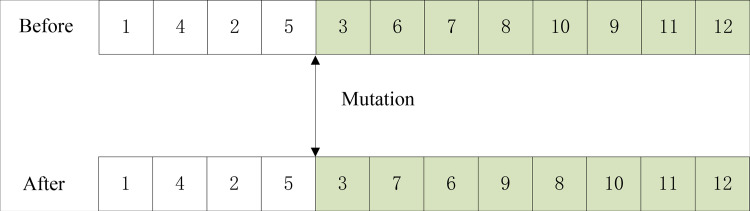
Gene comparison map before and after mutation.

To facilitate a clearer comparison of chromosome changes before and after mutation, juxtapose them together and present the comparison as depicted in Fig 9.

## 4 Experiments and analysis

Various objectives can be simultaneously optimized through a novel combined operation, offering a robust approach to addressing supply chain balance. Upon optimization, the optimal solution can be computed, representing the comprehensive optimum of the assembly line. The experiment was carried out by simulation and the experimental parameters were set manually. Thus, the actual supply cycle time of the supply chain should be selected as the main factor, and the work of each node should be considered, and then the operators of each node should be properly arranged, so as to reduce the labor cost. In the experiment, two different products are placed in the same supply chain. The product supply force includes 25 supply tasks, 13 nodes are allocated, the supply cycle time is 94s, and the demand of products A and B is 400 and 600 respectively. The experiments were done in the following environment: Ubuntu 18.10, Python 3.8.0, Intel i7-12700 CPU, NVIDIA Tesla V100 GPU.

### 4.1 Simulation process

In this study, the GA was employed to optimize the supply chain balance problem. The simulation was conducted within the following configuration: Ubuntu 18.10 operating system, Python 3.8.0 programming language, Intel i7-12700 CPU, and NVIDIA Tesla V100 GPU. The data inputs included 25 supply tasks distributed across 13 nodes, with a supply cycle time set at 94 seconds. The demand for products A and B was 400 and 600 units, respectively.

The GA process commenced with the initialization of a population comprising 100 individuals, where each individual represented a distinct configuration of task allocations and operator arrangements. The choice of population size significantly influences the algorithm’s exploratory capabilities and computational demands. Each individual was encoded using a combination of integer and binary coding to represent task distributions and operator assignments, respectively.

The fitness function was designed to evaluate the overall effectiveness of each solution, taking into account supply chain balance, labor costs, and product demand fulfillment. The fitness score was calculated as the sum of total costs, penalties for unmet demand, and penalties for supply chain imbalances.

For genetic operations, tournament selection was used, where 5 individuals were randomly selected from the population in each tournament, and the one with the highest fitness was chosen as the parent. Single-point crossover was applied to exchange gene segments between parent solutions, facilitating the exploration of new potential solutions and promoting genetic diversity. Additionally, mutation was introduced with a rate of 0.01, involving random changes in task allocations and operator arrangements to prevent convergence to local optima.

The GA was executed for a maximum of 100 generations or until convergence criteria were met. During each generation, the algorithm evaluated and evolved the population based on the fitness function, performing selection, crossover, and mutation to refine solutions. The termination criteria included reaching the maximum number of generations, achieving stability in fitness scores, or satisfying predefined objective standards.

Upon completion of the simulation, the optimal solution was identified from the final population, representing the most balanced and cost-effective configuration for the assembly line. The performance of this solution was analyzed based on total cost reduction, improvements in supply chain balance, and accuracy in meeting product demands. The results demonstrated the effectiveness of the GA approach in optimizing labor costs and enhancing supply chain balance, thereby validating its robustness in addressing complex supply chain challenges.

### 4.2 Comprehensive validity verification

The experimental design of the first part is considered to verify the effectiveness of the improved genetic algorithm, which is realized by showing the iterative process of the algorithm and the node assignment of actual verification. In iterative calculation, some individuals with high fitness value should be avoided from being screened out, and the crossover probability of individuals with high fitness value should usually be set. The parameters are shown in Table 2.

**Table 2:** Operator parameter setting of improved GA.

NP	Maxgen	$P_{c}$	$P_{m}$	$P_{c\min}$	$P_{c\max}$	$P_{m\min}$	$P_{m\max}$
100	100	0.8%	0.05%	0.5	0.9	0.005	0.01

**Fig 10:**
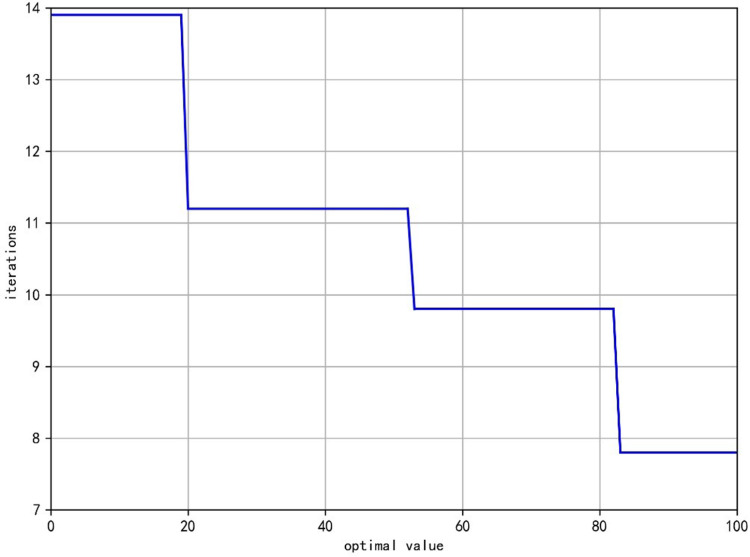
GA iteration graph.

The iterative process of the GA is illustrated in Fig 10, where the y-axis represents the number of iterations, and the x-axis denotes the optimal solution. The improved GA achieves the optimal solution after 83 iterations, reflecting the algorithm’s convergence speed and its feasibility for practical applications.

Tables 3 and 4 present the performance of the original GA and the improved GA in achieving multi-objective balance within the supply chain. These data highlight the model’s effectiveness in practical scenarios, particularly in terms of node scheduling and the associated economic and load variations.

**Table 3:** Original optimization results.

Node	Product	Number	Takt	Load	Cost	Time
1	2	1				
2	1,3	1				
3	5	1				
4	4,7,8	2				
5	6	1				
6	10,11	1				
7	9,14	1	94	0.678	1832	48
8	12	1				
9	13,16	1				
10	18	1				
11	15,17	1				
12	19,21,22,23	2				
13	20,24,25	2				

**Table 4:** Improved optimization results.

Node	Product	Number	Takt	Load	Cost	Time
1	2	1				
2	1,3	1				
3	5	1				
4	9	1				
5	6,10	1				
6	7,11	1				
7	13,16	1	94	0.535	1790	29.5
8	4,8	2				
9	12,14	1				
10	15,17	1				
11	18	1				
12	19,21,22,23	2				
13	20,24,25	2				

Upon comparing the two tables, notable differences emerge in the supply order of the multi-objective supply chain. The improved GA significantly outperforms the original GA, particularly in reducing the supply load of each node, The load decreases from an initial value of 0.678 to an improved value of 0.535. Additionally, labor costs are notably reduced, with the improved GA decreasing the cost from 1832 yuan to 1790 yuan.

Furthermore, the operational time of the improved GA shows a substantial reduction, decreasing from 48 seconds to just 29.5 seconds—a remarkable improvement in operational speed by approximately 39.5%. These compelling data conclusively demonstrate the efficacy and efficiency of the improved GA in addressing the complex optimization challenges of multi-objective supply chain management.

Overall, the improved GA offers more favorable supply order arrangements and achieves significant reductions in supply load, labor costs, and operational time. This makes it a highly effective solution for the multifaceted balancing optimization requirements within the multi-objective supply chain domain.

### 4.2 Node use

**Fig 11:**
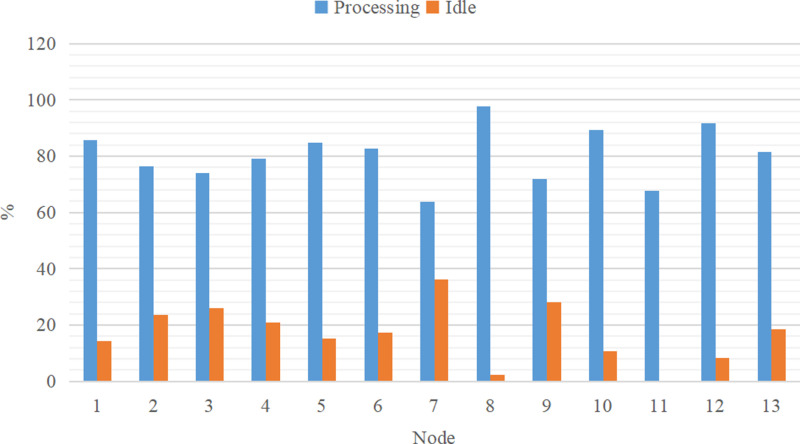
The state of each node before improvement.

**Fig 12:**
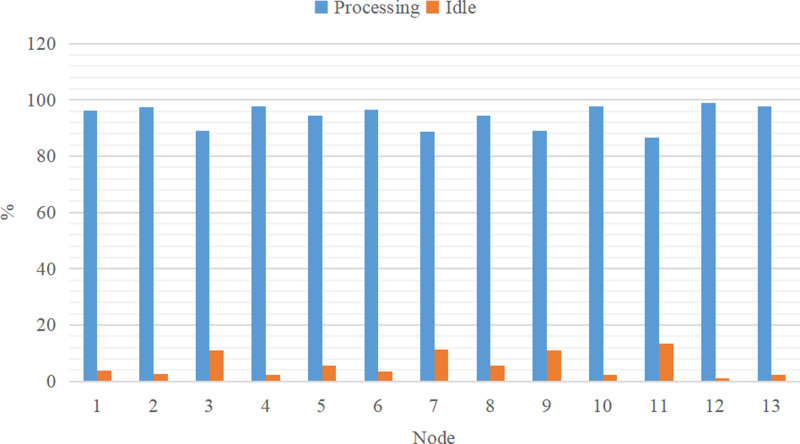
The state of each node after improvement.

In the previous Sections, I provided the theoretical foundation for enhancing the effectiveness of the genetic algorithm. In this section, I focus on the utilization of nodes in the simulation experiment. The experiments in Section 4.2 build upon the experiments conducted in Section 4.1, with code simulation is also employed. Following the simulation modeling, the status percentage reports of each node are presented in Fig 11 and Fig 12.

**Fig 13:**
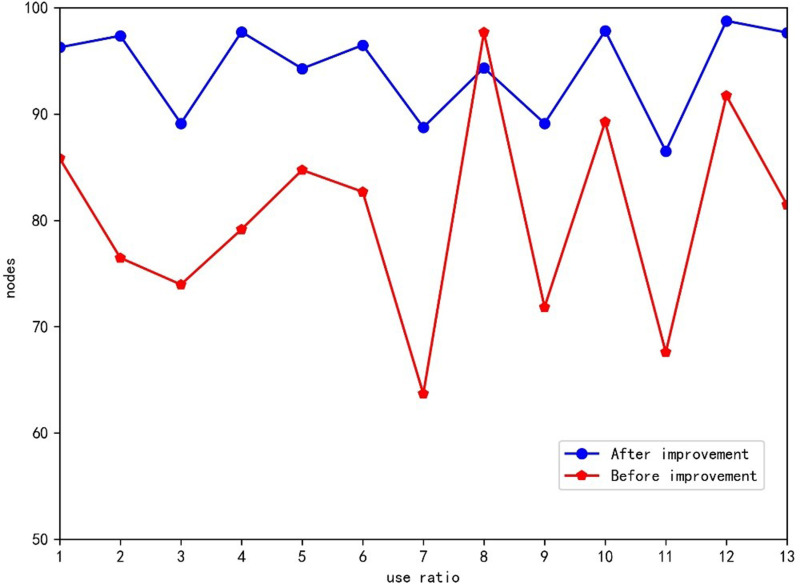
Comparison chart of use of each node.

To evaluate the utilization rates of the mixed-model assembly lines before and after the improvement, a comparison line chart as shown in Fig 13, was created based on Fig 11 and Fig 12. From Fig 13, it is evident that in the supply chain, the difference between the maximum and the minimum utilization rates across the first 13 nodes after improvement is 30.1%. This indicates that each node experiences significant idle time, leading to an incoherent supply process and suboptimal supply efficiency.

The utilization rate of each node varies greatly. For instance, when node 6 transitions to node 7, there is a sharp decline in utilization, resulting in considerable idle time at node 7. Similarly, the utilization rate of node 8 experiences a steep increase, causing a substantial backlog of products at node 8 and disrupting the overall supply chain balance.

However, in the supply chain optimized by the improved GA, although the number of nodes remains unchanged, the process arrangement at these nodes is modified. This adjustment leads to a significant reduction in the utilization ratio across the nodes, bringing it down to 12.25%. This marked improvement over the previous state demonstrates the proposed method’s ability to significantly enhance the scheduling efficiency of all resources within the supply chain, effectively conserving valuable resources.

Table 5 compared the Idle% (percentage of idle time) for different multi-objective optimization models, simulated for a range of nodes from 1 to 13. The values provided are hypothetical and intended to illustrate how each model’s performance might vary with increasing node counts.

**Table 5:** model comparison (Idle/%).

Nodes	The proposed model	NSGA-III	MOEA/D	MOPSO	HV-MOEA
1	3.73	12.50	12.00	13.00	11.50
2	2.64	11.80	11.50	12.50	10.80
3	10.88	11.20	11.00	12.00	10.30
4	2.28	10.80	10.60	11.50	10.00
5	5.73	10.50	10.30	11.00	9.80
6	3.52	10.30	10.00	10.80	9.60
7	11.26	10.10	9.80	10.50	9.40
8	5.66	9.90	9.60	10.30	9.20
9	10.88	11.70	111.40	11.10	12.00
10	2.17	9.50	9.20	9.90	8.80
11	13.49	19.30	19.00	117.70	18.60
12	1.24	9.10	8.80	9.50	8.40
13	2.34	8.90	8.60	9.30	8.20

NSGA-III (Non-dominated Sorting Genetic Algorithm III) [30]: NSGA-III is an advanced version of the NSGA-II algorithm designed to handle many-objective optimization problems (problems with more than three objectives). It introduces an external reference set to maintain diversity in the solution set.

MOEA/D (Multi-Objective Evolutionary Algorithm based on Decomposition) [31]: MOEA/D decomposes a multi-objective problem into a series of single-objective optimization problems and solves them concurrently. It is known for its efficiency and ability to handle a large number of objectives.

MOPSO (Multi-Objective Particle Swarm Optimization) [32]: MOPSO is a particle swarm optimization approach adapted for multi-objective problems. It maintains a diverse set of solutions and updates them using a swarm-based approach.

HV-MOEA (Hypervolume-Based Multi-Objective Evolutionary Algorithm) [33]: HV-MOEA focuses on optimizing the hypervolume indicator, which measures the volume covered by the Pareto front. This approach enhances the quality and diversity of the solution set by focusing on the hypervolume.

While the proposed model excels in minimizing idle time with fewer nodes, its performance becomes more variable with increased complexity. NSGA-III and MOEA/D offer consistent performance but do not match the proposed model’s efficiency in simpler cases. MOPSO shows relatively higher idle times, indicating less efficient resource utilization, while HV-MOEA stands out for its lowest idle percentages. The proposed model remains a competitive approach, particularly in less complex scenarios, and its performance highlights the need for further refinement to enhance stability across varying node counts.

## 5 Conclusion and future direction

By combining the principles of topological sorting theory with the random search method, I have refined my methodology to cultivate a diverse population, establishing inherent robustness in my approach. To optimize the crossover process, I have seamlessly integrated the elite selection method with the roulette approach. This synthesis embodies a harmonious blend of efficiency and elegance. Remarkably, the outcomes of my simulation experiment substantiate the efficacy of my innovative method. Specifically, I have achieved significant improvements. The utilization rate of the final node has been dramatically reduced from 30.10% to a mere 12.25%, while the load has shown a substantial decrease from 0.678 to 0.535. Additionally, the optimization of labor cost from 1832 to 1790 yuan underscores the remarkable efficiency of my approach. These exceptional results undeniably underscore the considerable enhancement in supply chain efficiency. Consequently, substantial savings in terms of manpower and scheduling time have been realized. By translating into practical applications, my model stands as a pioneering solution, ushering in a new era for digital industrial chains. This, in turn, injects a dynamic essence into the very fabric of economic development. Indeed, the simulation results in this study demonstrate a noticeable improvement in the utilization rate of each node. Nevertheless, some nodes still fall below the desired threshold of 90%, suggesting the potential areas for further enhancement in GA. To optimize its effectiveness, future schemes should consider incorporating additional parameters and fine-tuning the optimization process.

Despite the promising results achieved in my simulation, the proposed method exhibits certain limitations when applied to real-world scenarios. Firstly, the method’s reliance on topological sorting and random search may result in computational inefficiencies when scaled to larger, more complex supply chains. The elite selection combined with the roulette approach, while effective in our experiments, might not generalize well to situations where the diversity of the population is constrained or where the solution space is vast and highly variable. Moreover, the optimization of labor costs and node utilization rates, although impressive, may not fully account for the dynamic and unpredictable nature of real-world supply chains, where factors such as market volatility, supply chain disruptions, and evolving economic conditions could diminish the effectiveness of my approach.

Additionally, the current model’s performance in optimizing node utilization rates—while significantly improved—still leaves some nodes below the 90% threshold, indicating room for further refinement. This shortfall suggests that our approach may struggle to achieve optimal efficiency across all nodes, particularly in supply chains with highly irregular demand or in cases where certain nodes are critical bottlenecks.

Furthermore, the model’s efficacy in reducing labor costs might be less pronounced in industries where labor expenses are subject to fluctuations due to external factors such as wage increases, labor shortages, or regulatory changes. The model’s application to different industries or regions with varying labor market conditions could also limit its transferability and effectiveness.

## References

[1] Agrawal P, Narain R. Digital supply chain management: an overview. IOP Conf Ser: Mater Sci Eng. 2018;455:012074. doi: 10.1088/1757-899x/455/1/012074

[2] Dai Z. The optimization of costs of supply chain network. AMM. 2015;741:801–5. doi: 10.4028/www.scientific.net/amm.741.801

[3] Kitjacharoenchai P, Ventresca M, Moshref-Javadi M, Lee S, Tanchoco JMA, Brunese PA. Multiple traveling salesman problem with drones: mathematical model and heuristic approach. Comput Ind Eng. 2019;129:14–30. doi: 10.1016/j.cie.2019.01.020

[4] Sharma S, Khodadadi N, Saha AK, Gharehchopogh FS, Mirjalili S. Non-dominated sorting advanced butterfly optimization algorithm for multi-objective problems. J Bionic Eng. 2022;20(2):819–43. doi: 10.1007/s42235-022-00288-9

[5] Lu P, Ye L, Zhao Y, Dai B, Pei M, Tang Y. Review of meta-heuristic algorithms for wind power prediction: methodologies, applications and challenges. Appl Energy. 2021;301:117446. doi: 10.1016/j.apenergy.2021.117446

[6] Ayar M, Isazadeh A, Gharehchopogh FS, Seyedi M. NSICA: Multi-objective imperialist competitive algorithm for feature selection in arrhythmia diagnosis. Comput Biol Med. 2023;161:107025. doi: 10.1016/j.compbiomed.2023.107025 37245373

[7] Xue X, Chen J. Using compact evolutionary Tabu search algorithm for matching sensor ontologies. Swarm Evol Comput. 2019;48:25–30. doi: 10.1016/j.swevo.2019.03.007

[8] Morales-Castañeda B, Zaldívar D, Cuevas E, Maciel-Castillo O, Aranguren I, Fausto F. An improved simulated annealing algorithm based on ancient metallurgy techniques. Appl Soft Comput. 2019;84:105761. doi: 10.1016/j.asoc.2019.105761

[9] Katoch S, Chauhan SS, Kumar V. A review on genetic algorithm: past, present, and future. Multimed Tools Appl. 2021;80(5):8091–126. doi: 10.1007/s11042-020-10139-6 33162782 PMC7599983

[10] Garud KS, Jayaraj S, Lee M. A review on modeling of solar photovoltaic systems using artificial neural networks, fuzzy logic, genetic algorithm and hybrid models. Int J Energy Res. 2020;45(1):6–35. doi: 10.1002/er.5608

[11] Deng W, Zhang X, Zhou Y, Liu Y, Zhou X, Chen H, et al. An enhanced fast non-dominated solution sorting genetic algorithm for multi-objective problems. Inf Sci. 2022;585:441–53. doi: 10.1016/j.ins.2021.11.052

[12] Lee CKH. A review of applications of genetic algorithms in operations management. Eng Appl Artif Intell. 2018;76:1–12. doi: 10.1016/j.engappai.2018.08.011

[13] Metawa N, Hassan MK, Elhoseny M. Genetic algorithm based model for optimizing bank lending decisions. Expert Syst Appl. 2017;80:75–82. doi: 10.1016/j.eswa.2017.03.021

[14] Reddy GT, Reddy MPK, Lakshmanna K, Rajput DS, Kaluri R, Srivastava G. Hybrid genetic algorithm and a fuzzy logic classifier for heart disease diagnosis. Evol Intel. 2019;13(2):185–96. doi: 10.1007/s12065-019-00327-1

[15] Zhuang XG, Shi XS, Zhang PJ, Liu HB, Liu CM, Wang HF. New induced mutation genetic algorithm for spectral variables selection in near infrared spectroscopy. J Appl Spectrosc. 2020;87(2):260–6. doi: 10.1007/s10812-020-00994-4

[16] Maleki N, Zeinali Y, Niaki STA. A k-NN method for lung cancer prognosis with the use of a genetic algorithm for feature selection. Expert Syst Appl. 2021;164:113981. doi: 10.1016/j.eswa.2020.113981

[17] Bakdi A, Hentout A, Boutami H, Maoudj A, Hachour O, Bouzouia B. Optimal path planning and execution for mobile robots using genetic algorithm and adaptive fuzzy-logic control. Rob Auton Syst. 2017;89:95–109. doi: 10.1016/j.robot.2016.12.008

[18] Yu W, Li B, Jia H, Zhang M, Wang D. Application of multi-objective genetic algorithm to optimize energy efficiency and thermal comfort in building design. Energy Build. 2015;88:135–43. doi: 10.1016/j.enbuild.2014.11.063

[19] Dharma F, Shabrina S, Noviana A, Tahir M, Hendrastuty N, Wahyono W. Prediction of indonesian inflation rate using regression model based on genetic algorithms. J Online Inform. 2020;5(1):45–52. doi: 10.15575/join.v5i1.532

[20] Salimian M, Ghobaei‐Arani M, Shahidinejad A. Toward an autonomic approach for internet of things service placement using gray wolf optimization in the fog computing environment. Softw Pract Exp. 2021;51(8):1745–72. doi: 10.1002/spe.2986

[21] Guerrero C, Lera I, Juiz C. Genetic-based optimization in fog computing: current trends and research opportunities. Swarm Evol Comput. 2022;72:101094. doi: 10.1016/j.swevo.2022.101094

[22] Behera I, Sobhanayak S. Task scheduling optimization in heterogeneous cloud computing environments: a hybrid GA-GWO approach. J Parallel Distrib Comput. 2024;183:104766. doi: 10.1016/j.jpdc.2023.104766

[23] Saxena D, Singh A. K. Communication cost aware resource efficient load balancing (care-lb) framework for cloud datacenter. Recent Adv Comput Sci Commun. 2020;12:1–00.

[24] Satrio P, Mahlia TMI, Giannetti N, Saito K. Optimization of HVAC system energy consumption in a building using artificial neural network and multi-objective genetic algorithm. Sustain Energy Technol Assessments. 2019;35:48–57. doi: 10.1016/j.seta.2019.06.002

[25] Owais M, Osman MK. Complete hierarchical multi-objective genetic algorithm for transit network design problem. Expert Syst Appl. 2018;114:143–54. doi: 10.1016/j.eswa.2018.07.033

[26] May G, Stahl B, Taisch M, Prabhu V. Multi-objective genetic algorithm for energy-efficient job shop scheduling. Int J Prod Res. 2015;53(23):7071–89. doi: 10.1080/00207543.2015.1005248

[27] Li H, Xu B, Lu G, Du C, Huang N. Multi-objective optimization of PEM fuel cell by coupled significant variables recognition, surrogate models and a multi-objective genetic algorithm. Energy Convers Manag. 2021;236:114063. doi: 10.1016/j.enconman.2021.114063

[28] Lamini C, Benhlima S, Elbekri A. Genetic algorithm based approach for autonomous mobile robot path planning. Procedia Comput Sci. 2018;127:180–9. doi: 10.1016/j.procs.2018.01.113

[29] Rahmani Hosseinabadi AA, Vahidi J, Saemi B, Sangaiah AK, Elhoseny M. Extended genetic algorithm for solving open-shop scheduling problem. Soft Comput. 2018;23(13):5099–116. doi: 10.1007/s00500-018-3177-y

[30] Gu Q, Wang R, Xie H, Li X, Jiang S, Xiong N. Modified non-dominated sorting genetic algorithm III with fine final level selection. Appl Intell. 2021;51(7):4236–69. doi: 10.1007/s10489-020-02053-z

[31] García-Morales MA, Brambila-Hernández JA, Fraire-Huacuja HJ, Frausto-Solis J, Cruz-Reyes L, Gómez-Santillan CG, et al. Multi-objective evolutionary algorithm based on decomposition to solve the Bi-objective internet shopping optimization problem (MOEA/D-BIShOP). Mexican International Conference on Artificial Intelligence; 2023 Nov. Cham: Springer Nature Switzerland. p. 326–36.

[32] Han H, Liu Y, Hou Y, Qiao J. Multi-modal multi-objective particle swarm optimization with self-adjusting strategy. Inf Sci. 2023;629:580–98. doi: 10.1016/j.ins.2023.02.019

[33] Wen C, Ma H. A two-stage hypervolume-based evolutionary algorithm for many-objective optimization. Mathematics. 2023;11(20):4247. doi: 10.3390/math11204247

